# Injection Molding of 3-3 Hydroxyapatite Composites

**DOI:** 10.3390/ma13081907

**Published:** 2020-04-17

**Authors:** Jonas Biggemann, Patrizia Hoffmann, Ivaylo Hristov, Swantje Simon, Philipp Müller, Tobias Fey

**Affiliations:** 1 Department of Materials Science (Glass and Ceramics), University of Erlangen-Nuernberg, Martensstr. 5, D-91058 Erlangen, Germany; jonas.biggemann@fau.de (J.B.); patrizia.hoffmann@fau.de (P.H.); Ivaylo.Hristov@fau.de (I.H.); Swantje.Simon@fau.de (S.S.); philipp.georg.mueller@fau.de (P.M.); 2 Frontier Research Institute for Materials Science, Nagoya Institute of Technology, Gokiso-cho, Showa-ku, Nagoya 466-8555, Japan

**Keywords:** ceramic injection molding, sacrificial templating, porous hydroxyapatite, interpenetrating composites, bone grafts, dental implants

## Abstract

The manufacturing of ideal implants requires fabrication processes enabling an adjustment of the shape, porosity and pore sizes to the patient-specific defect. To meet these criteria novel porous hydroxyapatite (HAp) implants were manufactured by combining ceramic injection molding (CIM) with sacrificial templating. Varied amounts (Φ = 0–40 Vol%) of spherical pore formers with a size of 20 µm were added to a HAp-feedstock to generate well-defined porosities of 11.2–45.2 Vol% after thermal debinding and sintering. At pore former contents Φ ≥ 30 Vol% interconnected pore networks were formed. The investigated Young’s modulus and flexural strength decreased with increasing pore former content from 97.3 to 29.1 GPa and 69.0 to 13.0 MPa, agreeing well with a fitted power-law approach. Additionally, interpenetrating HAp/polymer composites were manufactured by infiltrating and afterwards curing of an urethane dimethacrylate-based (UDMA) monomer solution into the porous HAp ceramic preforms. The obtained stiffness (32–46 GPa) and Vickers hardness (1.2–2.1 GPa) of the HAp/UDMA composites were comparable to natural dentin, enamel and other polymer infiltrated ceramic network (PICN) materials. The combination of CIM and sacrificial templating facilitates a near-net shape manufacturing of complex shaped bone and dental implants, whose properties can be directly tailored by the amount, shape and size of the pore formers.

## 1. Introduction

The restoration of large bone defects represents one of the key issues in current orthopedic and trauma surgery [1,2]. Especially critical bone defects, arising from diseases (such as osteoporosis), tumor surgeries or fractures in all kinds of sizes and shapes, cannot naturally be self-repaired [3,4,5]. Unlimited and patient individualized restorations of those complex defects can only be provided by bone graft substitutes. To fulfill the biological and mechanical functions of bone, ideal bone substitutes require an interconnected pore network with adjustable pore sizes to promote bone ingrowth combined with a simultaneous sufficient mechanical strength to withstand mechanical loads [6,7,8]. The most promising materials are calcium phosphate-based ceramics (such as bioactive hydroxyapatite or bioresorbable β-tricalciumphosphate) due to their chemical similarity to apatite, the natural bone mineral, and the ability to form a strong bonding to natural bone [1]. To meet these biological and mechanical criteria, various fabrication techniques for porous hydroxyapatite (HAp) scaffolds were implemented including replica method [9,10,11,12], sacrificial templating [11,13,14,15], direct foaming [10,14,16], freeze casting [7,17], extrusion [18] and additive manufacturing [19,20,21,22]. However, not only the low mechanical strength and toughness of monolithic porous HAp limits the application as load-bearing implants [1], also the fabrication of complex-shaped implants with a controlled porosity on an industrial level remains so far unsolved. For that reason, bulk ceramic implants are still manufactured by subtractive computer-aided milling (CAD/CAM process) of biocompatible zirconia or alumina [23,24], while bioactive HAp is only used in coatings or for non-load-bearing bulk implants [25]. Thus, there is a high demand to establish manufacturing techniques for HAp bone and dental implants, which provide a near-net shaping of implants with complex geometries combined with an adjustable porosity to enable an industrial fabrication of ideal patient-specific implants.

Ceramic injection molding (CIM) is a cost-effective industrial processing technique for complex-shaped ceramic components with high dimensional accuracies [26,27,28]. Although CIM enables the fabrication of near-net shaped ceramic implants, the technique itself does not provide a simple and fast adjustability of the total porosity and pore sizes. So far, the porosity in porous CIM was either introduced by variation of the solid loading in the ceramic-wax-feedstock [27], partial sintering [26,29,30,31,32], direct foaming [33] or by using mixtures of coarse and fine raw powders [28]. Only for metal injection molding sacrificial templates have been used in the feedstock to generate well-defined porosities after the thermal heat treatment [34,35,36]. Sacrificial templating is the most straightforward manufacturing technique to tailor defined pore structures in a broad porosity range by changing the amounts (20–90 Vol%), shapes (such as spheres or fibers) and sizes (1-700 µm) of the used pore formers [37].

In this work, we present the fabrication of porous HAp implants with adjustable porosities and pore sizes by combining injection molding with sacrificial templating. Feedstocks for porous ceramics were fabricated by adding varied amounts (0–40 Vol%) of phenolic resin spheres as spherical pore formers to the HAp-wax-feedstocks. The resulting microstructural and mechanical properties were investigated in dependence of the pore former content. In addition, the properties of interpenetrating (3-3) ceramic-polymer composites, manufactured by infiltrating a urethane dimethacrylate (UDMA)-based monomer solution into the porous injection molded HAp preforms, will be discussed with regard to their application as potential dental implants.

## 2. Material and Methods

### 2.1. Injection Molding of Porous Hydroxyapatite Ceramics

**Figure 1:**
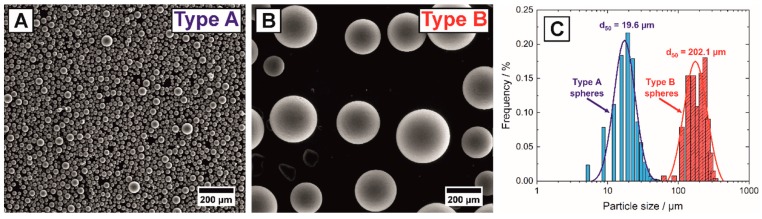
Microstructure (**A**,**B**) and particle size distributions (**C**) of the type A (d_50_ = 20 µm) and type B (d_50_ = 200 µm) phenolic resin spheres utilized as spherical pore formers.

Porous hydroxyapatite (HAp) ceramics were fabricated utilizing ceramic injection molding combined with the addition of sacrificial templates. Before use, the HAp raw powder (04238, Sigma-Aldrich Corp., St. Louis, MO, USA) was calcined at 1000 °C for 2 h and afterwards wet-milled in a planetary mill (Pulverisette 6, FRITSCH GmbH, Idar-Oberstein, Germany) with 500 rpm for 10 min in isopropanol using ZrO_2_ grinding balls (Ø = 2 mm, Tosoh Corp., Tokyo, Japan). After drying and sieving (35 mesh), a deagglomerated powder with a mean particle size of d_50_ = 1.8 µm and a specific surface area of S_v_ = 8.3 m²/g was obtained. The milled powder was hydrophobized in dry hexane (Merck KGaA, Darmstadt, Germany) using 0.65 mg/m² stearic acid (Honeywell Specialty Chemicals Seelze GmbH, Seelze, Germany) as surfactant. For the feedstock preparation the hydrophobized HAp powder was dispersed in molten paraffin (Granopent P, Carl Roth GmbH, Karlsruhe, Germany) and carnauba wax (naturfarben, Carl Roth GmbH, Karlsruhe, Germany) at 120 °C for 12 h under continuous stirring (RW 20 mixer, IKA-Werke GmbH & Co. KG, Staufen, Germany). The final HAp feedstock contained 50.0 Vol% hydrophobized HAp powder, 44.9 Vol% paraffin wax and 5.1 Vol% carnauba wax. Two kinds of phenolic resin spheres, type A (BRHF-035 Brace GmbH, Karlstein, Germany) and type B (BRHF-250 Brace GmbH, Karlstein, Germany) differing by one order of magnitude, were used as pore formers in this work. Figure 1 shows the microstructure of the two types of utilized pore formers and the corresponding particle size distributions. The particle size distributions were determined by image analysis of several SEM-micrographs for both types, analyzing more than 2000 spheres for the small type A and more than 250 spheres for large type B, respectively. Mean particle sizes of $d_{50}^{A}$ = 19.6 µm and $d_{50}^{B}$ = 202.1 µm were measured for type A and type B spherical pore formers, respectively. For an easier understanding and a uniform nomenclature, the two types of resin spheres will in the entire manuscript be classified by their size and called 20 µm (= type A) and 200 µm (= type B) pore formers.

Feedstocks for porous ceramics were fabricated by adding various amounts (0, 10, 20, 30 and 40 Vol%) of 20 µm pore formers to the 50 Vol% HAp feedstock. Additionally, a bimodal pore size distribution containing 15 Vol% of 20 µm and 5 Vol% of 200 µm spherical pore formers was realized. The pore former containing HAp-feedstocks were homogenized for an additional mixing time of 12 h before molding. The casting molds were manufactured utilizing a negative replica technique [29,38]. Positive polymer preforms were 3D-printed with a z-resolution of 30 µm with a SLA 3D-printer (Digitalwax^®^ 028J, DWS S.r.l., Thiene, VI, Italy) and afterwards molded with a PDMS elastomer (Elastosil M 4643 A/B, Wacker Chemie AG, München, Germany). Plates with dimensions of 35 mm × 35 mm × 4 mm and complex shaped facial, cranial and dental implants were 3D-printed and molded. The feedstocks were transfer molded into the negative PDMS forms at 120 °C. The binder was thermally removed by wick debinding in an Al_2_O_3_ nano-powder bed (AKP-50, Sumitomo Chemical Co. Ltd., Tokyo, Japan) with a d_50_ of 200 nm. Afterwards the samples were sintered at 1250 °C for 2 h with heating rates of 5 K·min^−1^.

### 2.2. Fabrication of HAp/UDMA Composites

HAp/UDMA composites were fabricated by infiltration of porous HAp preforms (3–3 HAp/air composites) with a liquid monomer mixture. As porous preforms, only HAp samples with 30 and 40 Vol% pore formers were used. For the monomer mixture, diurethane dimethacrylate (UDMA, Sigma Aldrich Corp., St. Louis, MO, USA) and tryethylene glycol dimethacrylate (TEGDMA, Sigma Aldrich Corp., St. Louis, MO, USA) were mixed in a molar ratio of 1:1. 0.15 wt% of benzoyl peroxide (Sigma Aldrich Corp., St. Louis, MO, USA) was added to the monomer solution, acting as thermal radical initiator for the polymerization reaction. The monomer solution was homogenized for 1 h on a magnetic stirrer. The monomer infiltration was performed inside a desiccator equipped with a pressure-equalizing dropping funnel, allowing a simultaneous degassing of the porous HAp samples and the monomer solution. The porous HAp plates were placed inside PDMS forms. Before infiltration the monomer solution and the porous HAp samples were degassed for 3 h with pressures <2500 Pa. The infiltration was performed by dropwise addition of the UDMA/TEGDMA solution through the valve until the samples were fully covered, maintaining the vacuum for an additional 3 h. After the infiltration, the samples were stored for 1 h at atmospheric pressure and afterwards polymerized at 130 °C for 3 h.

### 2.3. Characterization

For the mechanical testing of the porous HAp and HAp/UDMA composites, bending bars with dimensions 25 mm × 2.5 mm × 2 mm were manufactured by grinding off the excessive material with a high precision surface grinding machine (MPS 2 R300, G&N Genauigkeits Maschinenbau Nürnberg GmbH, Erlangen, Germany) and cutting with diamond wire saw (DWS175, Diamond WireTec GmbH & Co. KG, Weinheim, Germany). To determine the open porosity, the bulk and true density of all samples were determined by Archimedes’ principle according to DIN EN 623-2 in distilled H_2_O [39]. The total porosity was calculated from the geometric density by determining the dimensions and mass of the bending bars. The closed porosity was calculated from the difference between total and open porosity. The microstructure of fracture surfaces and polished cross-sections of both non-infiltrated and infiltrated samples was investigated by scanning electronic microscopy (SEM Quanta 200, FEI Deutschland GmbH, Frankfurt am Main, Germany). To visualize the interconnectivity of the pores and the resulting polymeric UDMA network the HAp matrix of the HAp/UDMA composites was completely etched in 32 wt% hydrochloric acid solution (Merck KGaA, Darmstadt, Germany) for 2 days. The microstructure of the etched polymer network was identically investigated. The surface-to-surface inter pore distance was determined from SEM micrographs of polished surfaces by image analysis using a plugin for nearest neighbor distance (NND) calculation of (FiJi) ImageJ. The 3D microstructure was additionally characterized by micro computer tomography (µCT, Skyscan 1172, Bruker Micro CT, Kontich, Belgium) with a resolution of 2.98 µm/voxel. The tungsten X-ray tube was operated with 80 kV and 100 mA with a wavelength of λ = 0.024 nm, an 11 MP detector and an Al filter were used. The samples were rotated for 180° with a rotation step size of 0.2°. The recorded 2D sinograms were reconstructed with NRecon (Version 1.6, Bruker Micro CT, Kontich, Belgium) and 3D visualized using the software Amira (Version 5.6, Thermo Fisher Scientific Inc., Waltham, MA, USA).

The Young’s moduli of the 25 mm × 2.5 mm × 2 mm bars were non-destructively measured by impulse excitation method according to DIN EN 843-2 and ASTM-E1876 using a condenser microphone (Audix TM-1, Audix Microphones, Wilsonville, OR, USA) and the software Buzz-o-sonic (Version 5.9, BuzzMac International LLC, Glendale, WI, USA) [40,41]. The flexural strengths were determined in 4-point-bending tests (Instron 5565, Instron GmbH, Pfungstadt, Germany) according to DIN EN 843-1 with a support span of 20 mm and a constant crosshead speed of 0.5 mm·min^−1^ [42].

## 3. Results and Discussion

### 3.1. Microstructural and Mechanical Properties of the Porous Injection Molded HAp

**Figure 2:**
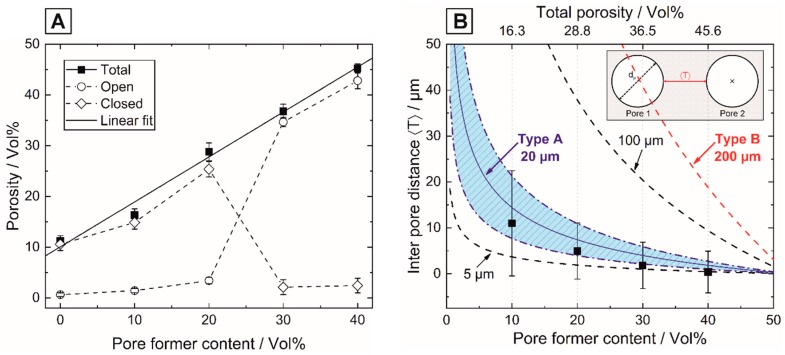
Porosity fractions (total, open and closed) of the porous hydroxyapatite (Hap) samples in dependence of the pore former content containing 20 µm spherical pore formers (**A**): The linear fit of (**A**) shows the linear relation (continuous line) between the total porosity and the pore former content, the dashed lines are only a guidance for the eye. (**B**) shows the dependence of the real surface-to-surface inter pore distance (data points represented by square symbols), determined by image analysis from SEM-micrographs, on the pore former content for the 20 µm spherical pore formers. The experimental data was compared to the model of Equation (1) inserting the measured particle size distribution of the 20 µm spheres of Figure 1C (see material and methods, here highlighted in the blue section) and for different pore sizes including 5, 100 and 200 µm (dashed lines).

After debinding and sintering (1250 °C, 2 h) of the HAp samples containing 0 to 40 Vol% of 20 µm spherical pore formers, total porosities between 11.2 and 45.2 Vol% were obtained. The residual sintering porosity of 11.2 ± 1.0 Vol% in the ceramic matrix without pore formers was considered the optimum between almost complete densification and phase purity. At higher temperatures (T ≥ 1300 °C) the thermal decomposition of HAp (Ca_10_(PO_4_)_6_(OH)_2_) to β-TCP (Ca_3_(PO_4_)_2_) and TTCP (Ca_4_(PO_4_)_2_O) was confirmed by XRD measurements (not shown here) in accordance with the literature [43], thus preventing the fabrication of dense and phase-pure HAp ceramics from a feedstock with 50 Vol% solid loading. Figure 2A shows the total porosity and the corresponding fractions of the open and closed porosity in dependence of the pore former content. A mean pore size of d_50_ = 22.5 µm and an average linear shrinkage of 18.3% ± 0.6% were determined, which both were found to be independent of the examined pore former content. The measured mean pore size agrees well with the initial pore former size of the 20 µm spheres.

**Figure 3:**
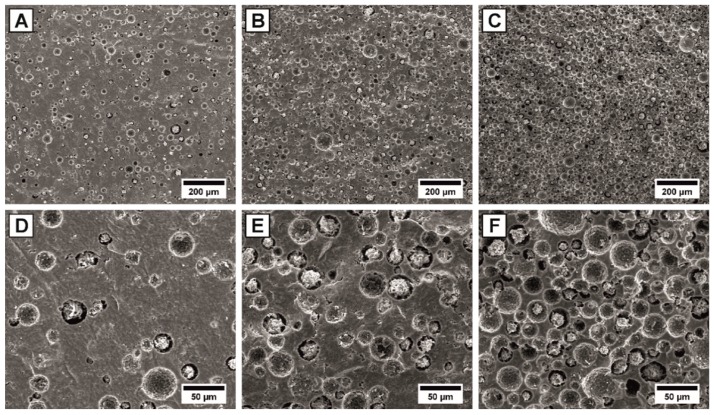
SEM-micrographs of fracture surfaces showing the microstructure of the porous HAp samples containing 10 (**A**,**D**), 20 (**B**,**E**) and 40 (**C**,**F**) Vol% of 20 µm spherical pore formers. The lower 250× magnification images of (**A**–**C**) display the homogeneous distribution of the pores, while the higher 1000× magnification images of (**D**–**F**) show the change of connectivity from isolated to interconnected pores.

The direct proportionality between the total porosity and the pore former content, shown by the linear regression (R² = 0.995) of Figure 2A, shows the free adjustability of the porosity by utilizing varied amounts of sacrificial templates as pore forming agents [37]. The corresponding microstructure of the 20 µm phenolic resin spheres containing samples are shown for a pore former content of 10, 20 and 40 Vol% in the SEM micrographs of Figure 3.

Homogeneous pore distributions were achieved independent from the examined pore former content, showing a good homogenization of the pore formers in the HAp-wax-suspensions due to the wet mixing process, Figure 3. Up to a volume content of 20 Vol% pore formers, the majority of the pores are isolated and thus closed, which was optically confirmed by the SEM micrographs of Figure 3B,E. At higher pore former contents (30 and 40 Vol%) cell windows between adjacent pores are formed and interconnected, open-porous networks with closed porosities less than 2.4 Vol% are generated, Figure 3C,F. Interconnected pore networks are mandatory to ensure the biological integration of implants associated with a complete vascularization and rapid bone ingrowth [44]. The transition from isolated closed pores to an interconnected, open pore network can be described by percolation models and the percolation threshold, representing the critical pore volume content [45,46,47,48]. The determination of the percolation threshold (Φ_c_) for interconnected pore networks is however extremely complex as it strongly depends on several pore parameters such as the shape [46,47], size [48], distribution [45] and packing density [49] and will therefore mainly be determined by the choice and dispersion of the utilized pore formers. For spherical pore networks a theoretical percolation threshold of Φ_c_ = 28.965 Vol% [47] is expected, agreeing well with the complete open porosity found in this work at a pore former content of 30 Vol% for 20 µm spherical pore formers. Additionally, the observed closed porosity at 20 Vol% and complete open porosity at 30 Vol% of the 20 µm pore formers is consistent with porous gelcasted Ti, where comparable pore former sizes were used [50]. For a reasonable estimation of the percolation threshold for differently sized pore formers, we propose to determine the mean surface-to-surface inter pore distance 〈T〉. The surface-to-surface interpore distance 〈T〉 of spherical pores (transferred from the interparticle distance of spherical inclusions in particle composites) is given by [51]:

(1) $$\langle T\rangle =\xi \;d_{p}{\left(\frac{\pi }{2\alpha \Phi }\right)}^{\frac{1}{D}}-d_{p}$$

where d_p_ is the mean pore size, Φ the pore former content, ξ and α are distributional constants and D describes the dimension of the system with Dϵ{1,2,3}. The real surface-to-surface interpore distance was determined by image analysis of the polished SEM-micrographs calculating the nearest neighbor distance (NND) between the individual pores. Figure 2B shows the experimental interpore distance 〈T〉 in dependence of the pore former content (Φ) in comparison to model of Equation (1) inserting ξ ≈ 1, α = 3 and D = 3 and different pore former sizes (d_p_). With increasing pore former content the experimentally determined interpore distance 〈T〉 decreases and reaches 0.4 µm for 40 Vol% pore formers approaching 0 within the standard deviations, Figure 2B. An acceptable agreement of the experimental determined 〈T〉 with the fitted model of Equation (1) was found, inserting the particle distribution of the 20 µm spheres (highlighted as the blue section of Figure 2B). A mean surface-to-surface interpore distance of 〈T〉≤0 describes vividly the formation of an interconnected pore network between adjacent pores. Mathematically, the zero point of Equation (1) (〈T〉=0) equals Φ_c_ = π/6 ≙ 52.36 Vol% independent from the pore former size d_p_ (inserting ξ ≈ 1, α = 3 and D = 3). For practical use, however, the approximation to the zero point of the simple Equation (1) for different d_p_ allows a reasonable theoretical estimation of the percolation threshold Φ_c_. As shown in Figure 2B, pore sizes of <20 µm show an asymptotic convergence to 〈T〉=0 approaching the zero point of Equation (1), while larger pore sizes such as 100 or the 200 µm spheres (type B) require a volume content of Φ_c_ = 52.36 Vol% to obtain an interconnected pore network. In Table 1, all mentioned physical properties of the porous HAp ceramics are summarized.

**Figure 4:**
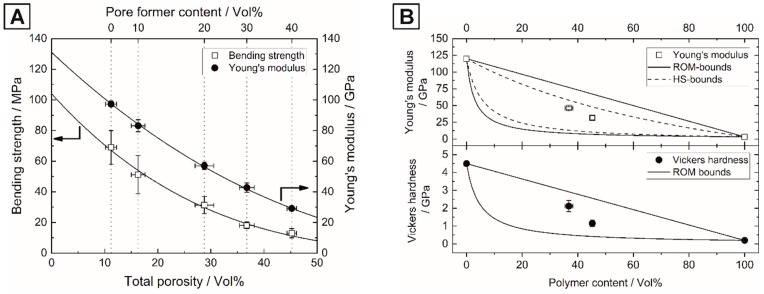
Mechanical properties of the porous HAp ceramics and HAp/urethane dimethacrylate (UDMA) composites: Young’s modulus and flexural strength of the porous HAp ceramics in dependence of the total porosity (**A**) and Young’s modulus and Vickers hardness of the HAp/UDMA composites in dependence of the UDMA-polymer content (**B**).

The mechanical properties of the porous HAp ceramics were investigated in terms of Young’s modulus and flexural strength by impulse excitation method and four-point bending. Figure 4A shows the mechanical properties as a function of the total porosity and pore former content. A general decrease in Young’s modulus and flexural strength was observed with increasing porosity. The Young’s modulus and flexural strength could be tailored in a broad range from 97.3 to 29.1 GPa and 69.0 to 13.0 MPa, respectively, by adding 0–40 Vol% of pore formers.

The porosity dependence of the Young’s modulus and flexural strength can be described by the well-established, semi-empirical power-law approach of Phani [52]:

(2) $$M_{p}=M_{0}\times {\left(1-\alpha \Phi_{p}\right)}^{n}$$

where the mechanical property (E, σ_f_) of the porous material M_p_ is given by the modulus of the dense material M_0_, the porosity (Φ_p_), the packing geometry factor α = 1–3.85 and the pore shape and orientation related exponent n = 2–7 [52,53]. A good agreement was obtained by fitting the model of Equation (2) to the experimental data for both Young’s modulus (R² = 0.9998) and flexural strength (R² = 0.9818) assuming α = 1. The mechanical properties of dense and porous HAp depend strongly on the powder synthesis and purity, the utilized fabrication technique and thus the resulting grain size and residual porosity, leading to great variations of reported literature values [1,54,55,56]. The determined E_0_ = 131 GPa is consistent with values for dense HAp $E_{0}^{*}$ = 80–140 GPa reported in the literature [54,55,56] and the corresponding n = 2.49 is close to the values for ideal isotropic materials with spherical pores n = 2–2.3 [57,58]. A stronger decrease was observed for the flexural strength with n = 3.69 and σ_0_ = 104 MPa, which agrees well with the typical values for commercial dense HAp $\sigma_{0}^{*}$ ≈ 100–120 MPa [1,56], although also much lower and higher values of 35 to 350 MPa have been reported [54]. The introduction of porosity allows a tailoring of the biological properties such as vascularization, bone ingrowth and resorption rates as well as the mechanical properties, like a reduction of the high stiffness compared to human bone to avoid stress shielding [59], however it is inevitably associated with a loss of mechanical strength. The fabrication of HAp-polymer composites is an attractive design approach to overcome these negative influencing factors and to further tailor the properties of future implants to the human body.

### 3.2. Microstructural and Mechanical Properties of the Interpenetrating HAp/UDMA Composites

The interconnected pore networks, formed by 30, 40 Vol% or higher amounts of pore formers incorporated in HAp ceramics, may serve as porous ceramic preforms for the fabrication of polymer infiltrated ceramic networks (PICNs), which belong to the class of interpenetrating (3–3) composites. Based on the advantages associated with the homogeneous, interconnected 3D distribution of both phases, PICNs have recently gained great interest to replace the widely used all-ceramic and resin-based ceramic particle-polymer (0–3) composites for dental restorations [60,61,62,63]. The properties of PICNs are more adapted to the hardness and stiffness of natural enamel and dentin than ceramics and in comparison to particle composites they exhibit a superior mechanical strength, stiffness, toughness and improved wear resistance [60,61,62,63]. In this work interpenetrating HAp/UDMA composites (IPCs) were manufactured by infiltrating a UDMA-TEGDMA monomer solution into the porous HAp preforms with 30 and 40 Vol% pore formers and a subsequent polymerization step. In Table 2, the microstructural and mechanical properties of the HAp/UDMA composites are summarized in reference to both pure materials.

**Figure 5:**
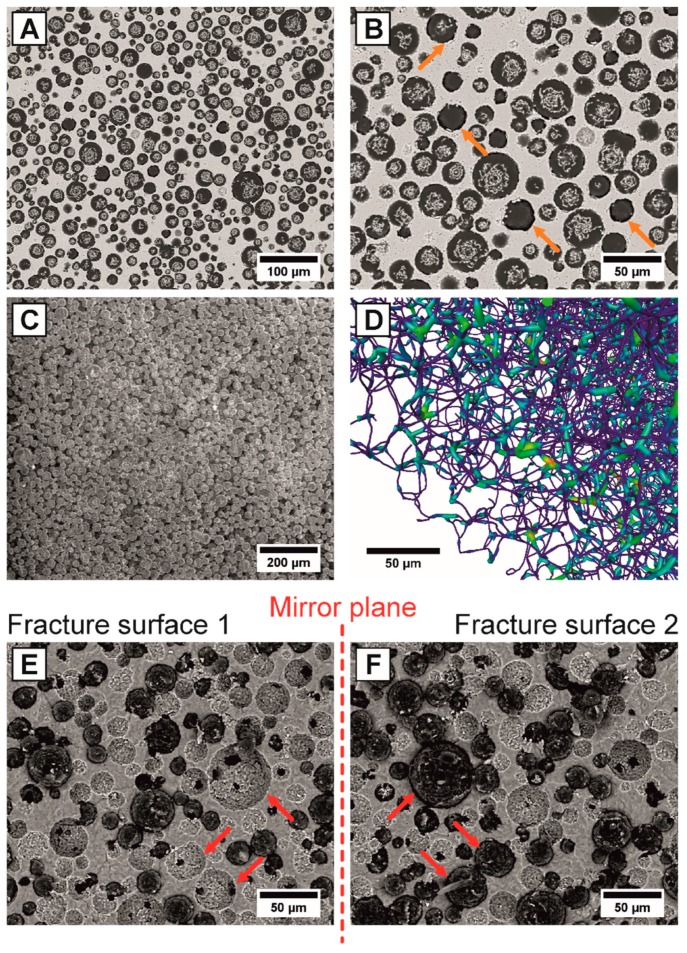
Microstructure of the fabricated HAp/UDMA composites with 40 Vol% of pore formers (**A**,**B**): SEM-micrographs show the complete polymer infiltration of all HAp pores (**A**) and polymerization shrinkage at the HAp/UDMA interface, highlighted by the orange arrows (**B**). Visualization of the interconnected UDMA-based polymer network (**C**,**D**): SEM-micrograph after etching-out the HAp ceramic matrix (**C**) and reconstructed, skeletonized µCT-polymer network indicating the pore sizes by a color heat-map with blue for pores >1 µm and red color >10 µm (**D**). Fracture behavior of the HAp/UDMA composites (**E**,**F**): The two fracture surfaces of one sample from the flexural testing, showing the fracture at the polymer/ceramic interface associated with 100% pullout effects of all polymeric spheres, highlighted as exemplary by three red arrows (**E**,**F**).

The SEM-micrographs of Figure 5A,B show the corresponding microstructure of the HAp/UDMA composites. Almost dense HAp/UDMA composites with theoretical densities of 97.6% and 94.9% were obtained for the porous HAp preforms containing 30 Vol% and 40 Vol% of pore formers, confirmed by the polymer-filled pores of Figure 5A. A complete infiltration (TD > 99%) could not be achieved based on the polymerization shrinkage of the monomer solution mixture, highlighted by the orange arrows of Figure 5A,B. For the pure UDMA/TEGDMA (1:1 mol) mixture a linear polymerization shrinkage of 9.0% ± 0.1% was measured, which is within the values reported of the pure constituents (UDMA: 8.7% ± 0.03% and TEGDMA: 14.1% ± 0.32%) [64]. Assuming a complete infiltration (100%) of the total pore volume for both preforms, the determined polymerization shrinkage of 9.0% causes porosities of 3.3% and 4.1% for an isotropic shrinkage, agreeing well with the measured theoretical densities (Table 2). To achieve fully densified PICN composites a pressure-assisted polymerization with pressures up to 300 MPa have to be applied additionally to the usage of coupling agents to improve the adhesion between polymer and ceramic [63,65,66]. However, in contrast to the small pore sizes (~1 µm) of typical PICN ceramic preforms fabricated by partial sintering, the utilized pore formers (~20 µm) of this work enable a significant decrease of the required infiltration pressure. The infiltration pressure for a cylindric pore with a radius r can be estimated by using the Hagen–Poiseuille law, where the applied pressure difference ∆p is proportional to 1/r^4^ [67]. Under identical test conditions, the infiltration pressure of ∆p_1_ = 300 MPa can be reduced to ∆p_2_ = 2 × 10^−3^ MPa by increasing the pore size from d_1_ = 1 µm to d_2_ = 20 µm (using ∆p_2_ = (r_1_/r_2_)^4^ × ∆p_1_), showing that for the simplified model no external pressure is required to infiltrate the porous HAp preforms. Regardless of that fact, an interconnected, cohesive polymeric network consisting of interconnected UDMA-based spheres was successfully realized by pressureless infiltration, as shown in the etched SEM-micrograph of Figure 5C and the µCT-derived reconstructed, skeletonized polymer network of Figure 5D. The color heat-map of the skeletonized polymer network represents the pore sizes with blue for pores >1 µm and red for pores >10 µm. A highly interconnected polymer network was obtained, in which the spherical pore formers (>10 µm) were connected via small cell windows, for which the size was between 1 and 10 µm.

The mechanical properties of the composites were investigated in dependence of the infiltrated polymer content, Figure 4B. The rule of mixtures (ROM) of Reuss and Voigt were used to theoretically describe the influence of the phase contents on the hardness and Young’s modulus, Equation (3) [68,69]. Additionally, the lower and upper Hashin–Shtrikman (HS) bounds were applied to more precisely model the experimental data of the Young’s modulus, Equation (4) [70,71]. For the pure reference materials, the values shown in Table 2 were used to fit the ROM- and HS-bounds.

(3) $$M_{c}=\Phi_{A}M_{A}+\left(1-\Phi_{A}\right)M_{B},\;\;M_{C}={\left(\frac{\Phi_{A}}{M_{A}}+\frac{\left(1-\Phi_{A}\right)}{M_{B}}\right)}^{-1}$$

(4) $$E_{C,upper}=\frac{E_{A}\left(\Phi_{A}E_{A}+\left(2-\Phi_{A}\right)\;E_{B}\right)}{\Phi_{A}E_{B}+(2-\Phi_{A})\;E_{A}},\;\;E_{C,lower}=\frac{E_{B}\left((1-\Phi_{A}\right)E_{B}+\left(1+\Phi_{A}\right)\;E_{A})}{(1-\Phi_{A})E_{A}+(1+\Phi_{A})\;E_{B}}$$

The mechanical properties M_c_ (or E_c_, respectively) of the composite are given by the volume fraction Φ and the mechanical property M_i_ (here Young’s modulus and Vickers hardness) of the constituents A and B. A general decrease of both Young’s modulus and Vickers hardness with increasing polymer content was observed. All experimental data points were within the calculated ROM and HS-bounds, agreeing well with the models of Equations (3) and (4) for hardness and Young’s modulus. However, the HAp/UDMA composites exhibited a lower flexural strength than the pure components (Table 2), which can be attributed to the poor interface adhesion caused by the discussed polymerization shrinkage. Nevertheless, the polymer infiltration lead to a significant increase in the flexural strength of +64% (to 29.6 ± 5.5 MPa) and +52% (to 19.7 ± 3.9 MPa) in comparison to the porous Hap that preforms with 30 and 40 Vol% of pore formers, respectively. Figure 5E,F shows the two fracture surfaces of one HAp/UDMA composite with 40 Vol% pore formers highlighting the typical fracture behavior of the composites. The fracture always occurred at the polymer-ceramic interface leading to complete pullout of all polymeric spheres (100% pull-out), shown in the mirrored fracture surfaces. Pullout effects in weak-interface composites are one of the most promising crack deflection mechanisms dissipating high crack energies, which can enhance the fracture toughness and crack resistance [72,73].

### 3.3. Potentials and Future Perspectives of the Porous HAp Ceramics and HAp/UDMA Composites

Ceramic injection molding (CIM) coupled with sacrificial templating facilitates the manufacturing of porous ceramics with complex geometries, tailored pore sizes and porosities, which may serve as restorative bone implants for critical bone defects. The microstructural and mechanical properties are directly determined by the amount, size and shape of the utilized pore former [37]. The mechanical properties of the porous HAp ceramics and interpenetrating HAp/UDMA composites are shown in Table 3 in comparison to human bone, teeth and other UDMA-based PICNs. For the used 20 µm spherical pore formers, the monolithic porous HAp ceramics exhibit mechanical properties between trabecular and cortical bone.

**Figure 6:**
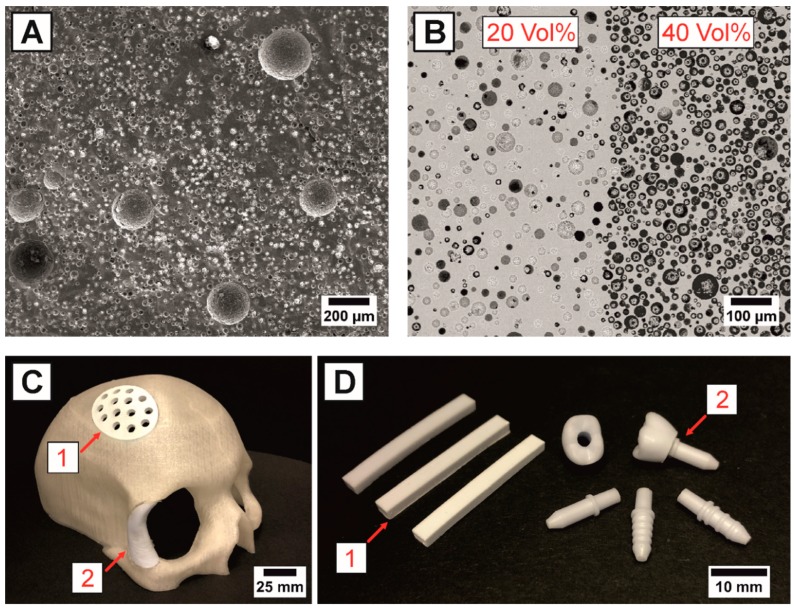
Future perspectives for porous HAp ceramics and HAp/UDMA composites fabricated by porous injection molding: Microstructure of a porous HAp sample with a bimodal pore size distribution containing 5 Vol% of 200 µm and 15 Vol% of 20 µm spherical pore formers (**A**). Polished SEM-micrograph of a HAp sample with a graded porosity, showing the defect-free interface between two layers containing 20 Vol% and 40 Vol% of 20 µm spherical pore formers (**B**). Potential non-load bearing cranial (1) and maxillofacial (2) implants for the porous HAp ceramics (**C**). Fabricated porous HAp bending bars with 0, 10 and 30 Vol% of pore formers (1) and potential two-part dental implants for the interpenetrating HAp/UDMA composites (2), showing a ceramic crown and three types of abutments with different anchoring geometries (**D**).

Based on the poor fracture toughness of monolithic HAp (~1.0 MPa·m^0.5^) [2,43], the porous HAp ceramics of this work can only be applied for non-load bearing applications. The combination of CIM and the 3D-printing process for the mold fabrication allows a flexible, near-net shape manufacturing of complex shaped implants. The patient-specific defect information including exact shape, size and surrounding bone density can be obtained from CT-scans, which afterwards can be implemented in the 3D-printing CAD files to generate patient individualized implants. Figure 6C shows exemplary fabricated cranial and maxillofacial HAp implants, which are inserted in a 3D-printed skull model. For the implant fabrication, the sintering shrinkage has to be considered, which with 18.3% ± 0.6% was found to be independent of the examined pore former content. The density, strength and stiffness of the HAp implants (Figure 4A) can then easily be adjusted to the age- and gender-related defect-surrounding bone density by changing the amounts of the utilized pore formers to avoid stress shielding effects. Although interconnected pore networks consisting of 20 µm spherical pore formers were successfully generated, for an efficient vascularization and bone ingrowth larger pore formers >250 µm have to be used [2]. To highlight the microstructural flexibility of the utilized fabrication technique, porous HAp ceramics with bimodal pore size distributions containing 5 Vol% of 200 µm and 15 Vol% of 20 µm spheres were fabricated, Figure 6A. As shown in Figure 6A, crack-free samples with homogeneously distributed bimodal pores, which differ by one order of magnitude, were successfully fabricated. Moreover, the technique is not only limited to the fabrication of homogenously distributed samples, but also enables designing of ceramics with a graded porosity. Figure 6B shows the defect-free interface of a graded porous HAp sample with 20 Vol% (left) and 40 Vol% (right) spherical pore formers (20 µm). Bioactive ceramics with graded porosity are the next generation biomaterials in tissue engineering to overcome the poor mechanical properties of monolithic porous ceramics (such as HAp) and to improve the biological integration by an enhanced nutrient transfer and osteogenic differentiation [76,77,78].

The properties of the porous injection molded HAp ceramics can further be tailored by combining the stiff ceramic matrix with a comparable soft UDMA-based polymer to form an interpenetrating composite, achieving properties, which cannot be obtained with the pure constituents, Table 2. Although the poor flexural strength of the HAp/UDMA composites requires future improvements in the manufacturing process (utilizing high pressure and temperature polymerization and coupling agents [63,65,66]), the achieved stiffness and hardness are comparable to natural enamel, dentin and other (commercial) PICNs and thus do not require further improvements for these types of applications [64,66,74,75], Table 2 and Table 3. The adaption of the stiffness and hardness to the properties of natural teeth is essential to avoid stress shielding effects and excessive wear of the antagonist [65]. In comparison to other PICNs, an additional advantage of HAp/UDMA material systems arises from the chemical similarity of HAp to the natural bone and teeth mineral providing strong bonding to surrounding bone tissue (osteoconduction) and therefore an enhanced mechanical anchoring of the implant. Restorative dental implants are commonly fabricated via subtractive CAD/CAM machining providing high accuracies but also high material waste, which cannot easily be recycled [23,24,65]. The utilized porous injection molding has great potential to design near-net shaped dental implants, which afterwards can be processed to PICNs by liquid polymer infiltration, to reduce processing steps and waste.

## 4. Conclusions

In this work, we demonstrated the fabrication of porous hydroxyapatite ceramics with well-defined porosities and pore sizes by combining ceramic injection molding with the addition of sacrificial templates. The novelty of this fabrication technique is the near-net shape manufacturing of complex shaped bone grafts with controlled porosity and connectivity. Their microstructural and mechanical properties can be directly adjusted to the age- and gender-related defect-surrounding bone density of patients by changing the amount, size and shape of the utilized pore formers to avoid stress shielding effects. The inherent brittleness of the monolithic HAp limits the usage to non-load bearing applications (e.g., cranial or maxillofacial implants). We demonstrated the feasibility of manufacturing interpenetrating HAp/UDMA composites with tremendous potential for use as restorative dental implants. Based on the chemical and physical similarity of HAp to the natural bone and tooth mineral, rejection reactions and excessive wear of the antagonists may be prevented. Although up to now the determined flexural strength requires further optimizations, the investigated Young’s modulus and hardness were comparable to natural dentin, enamel and other already commercialized polymer infiltrated network (PICN) materials and meet the requirements of the medical profession. However, in contrast to the commonly used subtractive CAD/CAM machining of PICNs, the here presented near-net shape fabrication technique does not require further post processing and thus does not produce waste as the complex dental implants can be directly obtained via the ceramic injection molding and afterwards can be processed to PICNs.

## Figures and Tables

**Table 1:** Physical properties of the porous HAp ceramics containing 0–40 Vol% of 20 µm spherical pore formers.

Pore Former Content /Vol%	Open /Vol%	Porosity (Φ_p_) Closed /Vol%	Total /Vol%	Interpore Distance 〈T〉 /µm	Young’s Modulus (E) /GPa	Flexural Strength (σ_f_) /MPa
0	0.6 ± 0.4	10.6 ± 1.2	11.2 ± 1.0	-	97.3 ± 1.9	69.0 ± 10.9
10	1.4 ± 0.4	14.9 ± 1.3	16.3 ± 1.3	11	83.1 ± 3.9	51.1 ± 12.5
20	3.4 ± 0.7	25.4 ± 1.5	28.8 ± 1.7	4.9	57.0 ± 2.5	31.4 ± 5.6
30	34.7 ± 0.9	2.1 ± 1.5	36.8 ± 1.4	1.8	42.8 ± 3.1	18.1 ± 2.3
40	42.8 ± 1.6	2.4 ± 1.4	45.2 ± 0.9	0.4	29.1 ± 0.9	13.0 ± 3.0

**Table 2:** Microstructural and mechanical properties of the interpenetrating HAp/UDMA composites and pure reference materials.

Samples	Preform Porosity	Geometric Density	Theoretical Density	Vickers Hardness	Young’s Modulus	Flexural Strength
	/Vol%	/g·cm^−3^	/%	HV1/GPa	/GPa	/MPa
Pure HAp	-	2.81 ± 0.05	88.8	4.5*	131**	104**
30 Vol% Inf.	36.8 ± 1.4	2.29 ± 0.05	97.6	2.12 ± 0.31	46.1 ± 2.1	29.6 ± 5.5
40 Vol% Inf.	45.2 ± 0.9	2.10 ± 0.04	94.9	1.15 ± 0.18	31.8 ± 3.3	19.7 ± 3.9
Pure UDMA/TEGDMA	-	1.20 ± 0.01	-	0.2 ± 0.04	3.9 ± 0.3	97.3 ± 21.4

* Data taken from literature [8], ** E_0_ and σ_0_ were extrapolated for dense HAp from Equation (2).

**Table 3:** Mechanical properties of the monolithic porous HAp ceramics and interpenetrating HAp/UDMA composites in comparison to human bone, teeth and UDMA-based polymer infiltrated ceramic networks (PICNs).

Monolithic Ceramic	Material	Porosity	Young’s Modulus	Vickers Hardness	Flexural Strength	Reference
		/Vol%	/GPa	/GPa	/MPa	
**Bone restoration**	Cortical Bone	5–10	7–30	-	50–150	[8,43,74]
	Trabecular bone	75–95	0.05–0.5	-	10–20	[43,74]
	Porous HAp	11.2–45.2	29–97	-	13–69	This work
**PICN**	**Material**	**Polymer Content**	**Young’s Modulus**	**Vickers Hardness**	**Flexural Strength**	**Reference**
		**/Vol%**	**/GPa**	**/GPa**	**/MPa**	
**Dental restoration**	Enamel	-	48–105	3–5.3	76	[74,75]
	Dentin	-	11–20.3	0.5–1.0	245–268	[74,75]
	HAp/UDMA*^,a^	37–45	32–46	1.2–2.1	20–30	This work
	Feldspar/UDMA**^,a^	28–41	16–28	1.1–2.1	131–160	[65]
	ZrO_2_/UDMA**^,a^	8–42	15–101	0.4–10.8	58–212	[66]

* pressureless polymerization, ** high pressure, high temperature polymerization, ^a^ excluding the properties of the pure constituents.
